# Supplementary material for: Subthalamic nucleus stabilizes movements by reducing neural spike variability in monkey basal ganglia
Source: Nat Commun. 2022 Apr 25;13:2233. doi: 10.1038/s41467-022-29750-2 (PMC9038919; doi:10.1038/s41467-022-29750-2)
Supplement: Supplementary file 1 — Supplementary Information [file 41467_2022_29750_MOESM1_ESM.pdf]

Subthalamic nucleus stabilizes movements by reducing neural spike variability in monkey basal ganglia

Authors: Taku Hasegawa, Satomi Chiken, Kenta Kobayashi, Atsushi Nambu*

*Correspondence: nambu@nips.ac.jp

Supplementary Information

List of Contents

Supplementary Figures 1-14
Supplementary Table 1
Supplementary References

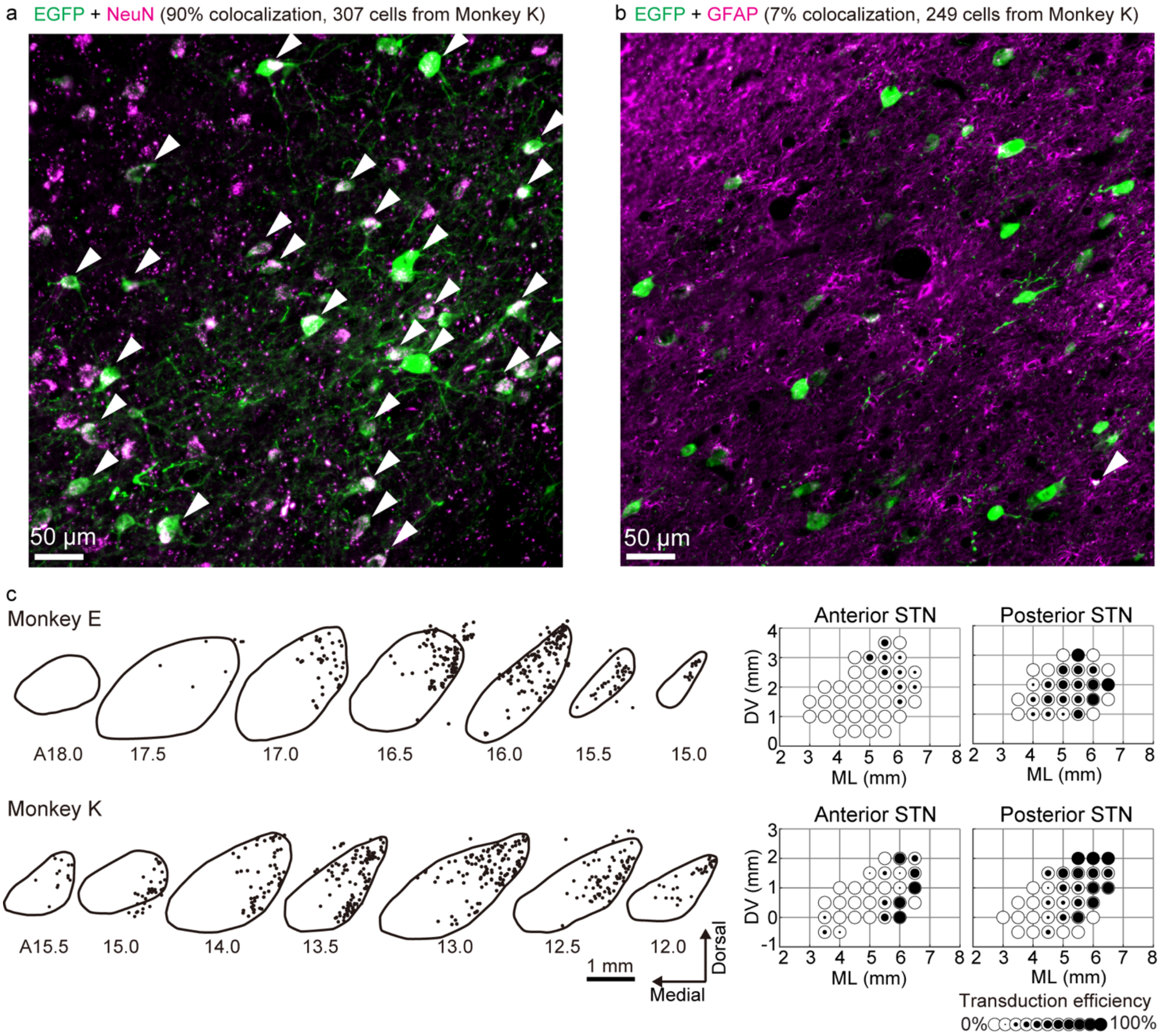

Supplementary Fig. 1 | Selectivity and distribution of transduced cells within the STN. a, Examination of transduction preference of AAV to neurons on brain slices from monkey K. Brain slices were double-immunostained with anti-GFP (green) and anti-NeuN (magenta) antibodies. Their colocalization, defined as the ratio of NeuN-positive cells among all GFP-positive cells, was 90% (307 cells from 7 brain slices). **b**, Same as **(a)** but for astrocytes using anti-GFP (green) and anti-GFAP (magenta) antibodies. Colocalization of GFP and GFAP was 7% (249 cells from 10 brain slices). **c**, Histologic examination of the STN in 7 frontal sections of monkeys E and K with the distance (mm) anterior to the origin of stereotactic coordinates (10 mm dorsal to the mid-interaural point). Cells labeled with a neuronal marker, NeuN, and anti-GFP antibody were considered transduced neurons with AAV and indicated by black dots (left). Frontal sections were divided into 0.5 mm × 0.5 mm squares along the dorsal-ventral (DV) and medial-lateral (ML) axes. Numbers of transduced neurons and NeuN-positive cells were counted, and transduction efficiencies, ratios of transduced neurons among all NeuN-positive cells, were calculated at each square. Transduction efficiencies were averaged along the anterior-posterior axis separately in anterior (3 sections) and posterior (4 section) STN regions and plotted along the DV and ML axes (right). Transduction efficiencies were also averaged in the dorsolateral part (DV ≥ 2, ML ≥ 5 in monkey E; DV ≥ 1, ML ≥ 5 in monkey K) of the posterior STN. The immunostaining was repeated twice (**a**) and three times (**b**) with similar results.

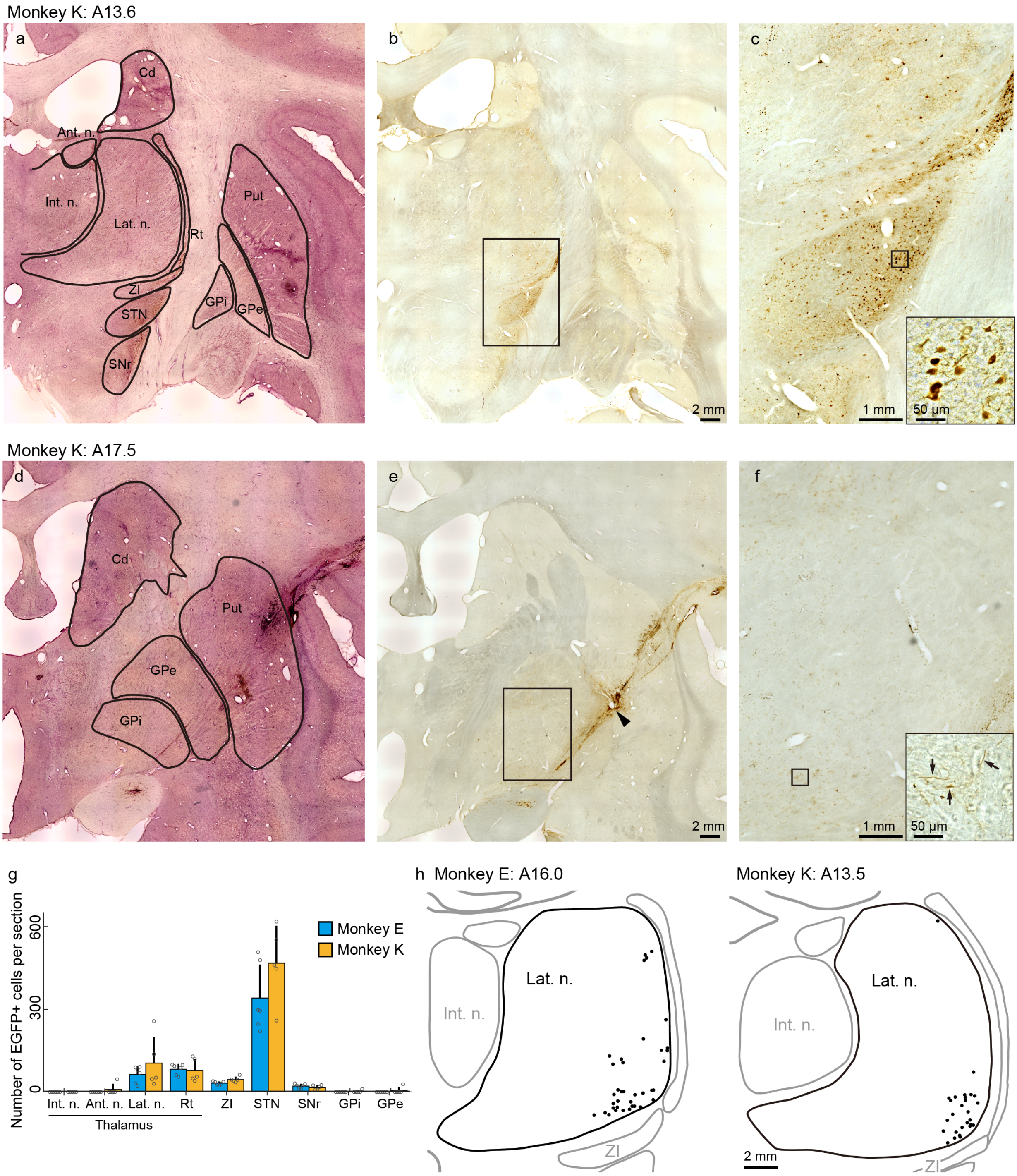

Supplementary Fig. 2 | Histologic examination for the distribution of transduced cells. a, Coronal section from monkey K with neutral red staining. **b**, EGFP-positive cells on the coronal section adjacent to **(a)**, visualized by an avidin-biotin-diaminobenzidine procedure. **c**, Magnified image of the rectangular area in **(b)**. Inset shows further magnification for the region indicated by a square. A small fraction of transduced cells were found dorsal to the STN such as the lateral nuclei of the thalamus (Lat. n.), thalamic reticular nucleus (Rt), and zona incerta (ZI). **d-f**, Same as **a-c** but for more anterior region where GPe/GPi neurons were recorded. The arrowhead in **e** indicates the site of electrolytic lesion. EGFP expression was found in the cell bodies of the STN (**c**) and putative axonal terminals in the GPe/GPi (**f,** arrows). However, hM4Di could be localized differently from

EGFP within a cell, since hM4Di was presumably cleaved from EGFP by 2A self-cleaving peptide. Transduced cells were rarely found in the GPe/GPi (**f**). **g**, Number of transduced cells in each brain region from 6 and 5 brain sections for monkeys E and K, respectively. Data are shown as mean ± SD. **h,** Distribution of transduced cells within the Lat. n. Transduced cells in the densest section were plotted, and those outside the Lat. n. were omitted for clarity. They were found in the most ventrolateral part of the Lat. n. and avoided its central part presumably representing the forelimb based on the somatotopic organization of the thalamus along its mediolateral axis[1–3]. The immunostaining was repeated twice with similar results (**b, c**, **e, f**). Ant. n., anterior nuclei of the thalamus; Int. n., intralaminar nuclei of the thalamus; SNr, substantia nigra pars reticulata; Cd, caudate nucleus; Put, putamen.

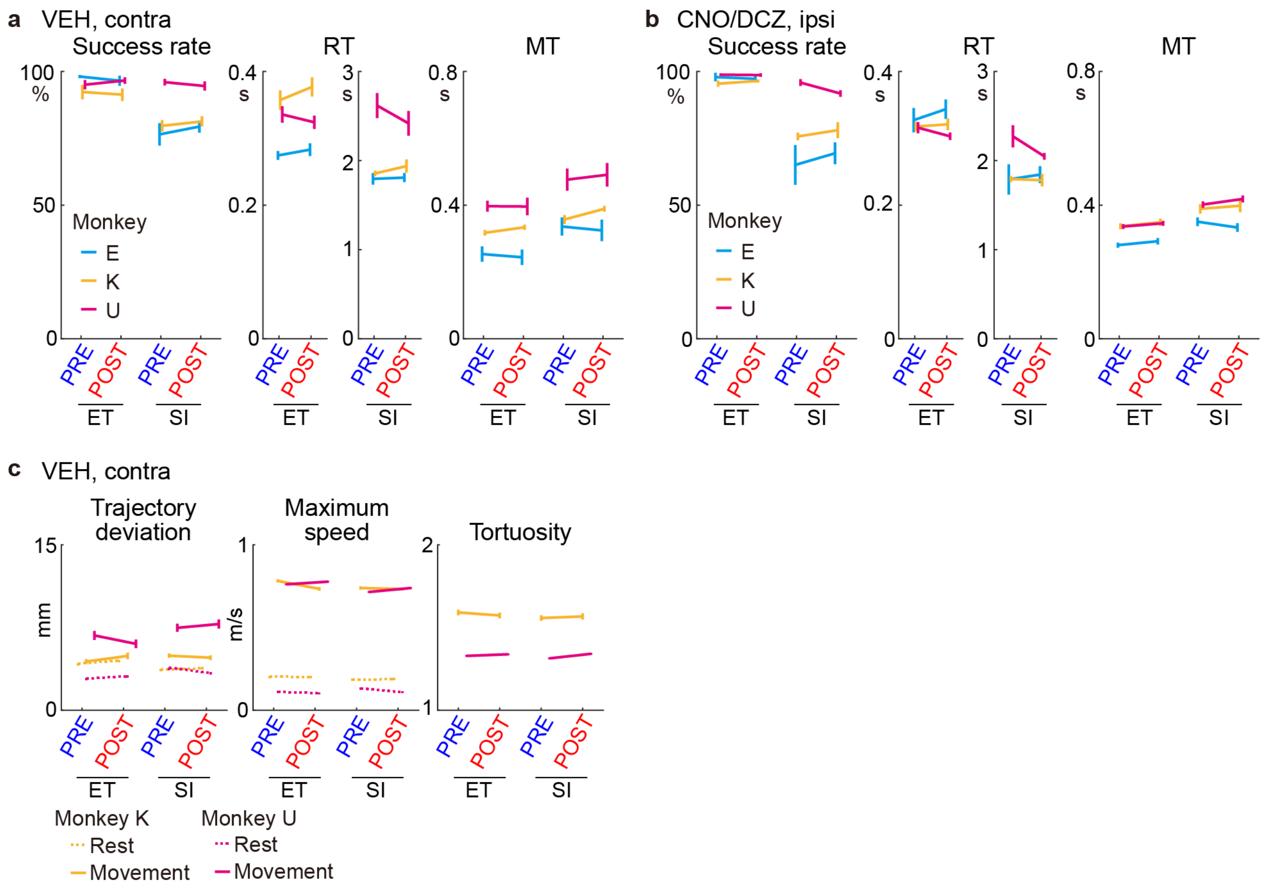

Supplementary Fig. 3 | Control experiments for DREADD ligand administration. a, **b**, Success rate, RT, and MT in control experiments, corresponding to Figure 2b. **a**, Performance of the task with VEH administration. The hand contralateral ('contra') to the AAV injection side was used. Error bars indicate SEM. No significant changes in the two-tailed Wilcoxon signed rank test (n = 8, 12, and 10 sessions for monkeys E, K, and U, respectively). **b**, Performance of the task using the ipsilateral hand ('ipsi') with CNO/DCZ administration. Error bars indicate SEM. No significant changes in the two-tailed Wilcoxon signed rank test (n = 8, 9, and 9 sessions). **c**, Analyses of wrist trajectories for monkeys K and U with VEH administration, corresponding to Figure 2c. Error bars indicate SEM. No significant changes in the two-tailed Mann-Whitney *U* test (monkey K, 74 ET and 70 SI trials; monkey U, 45 ET and 54 SI trials).

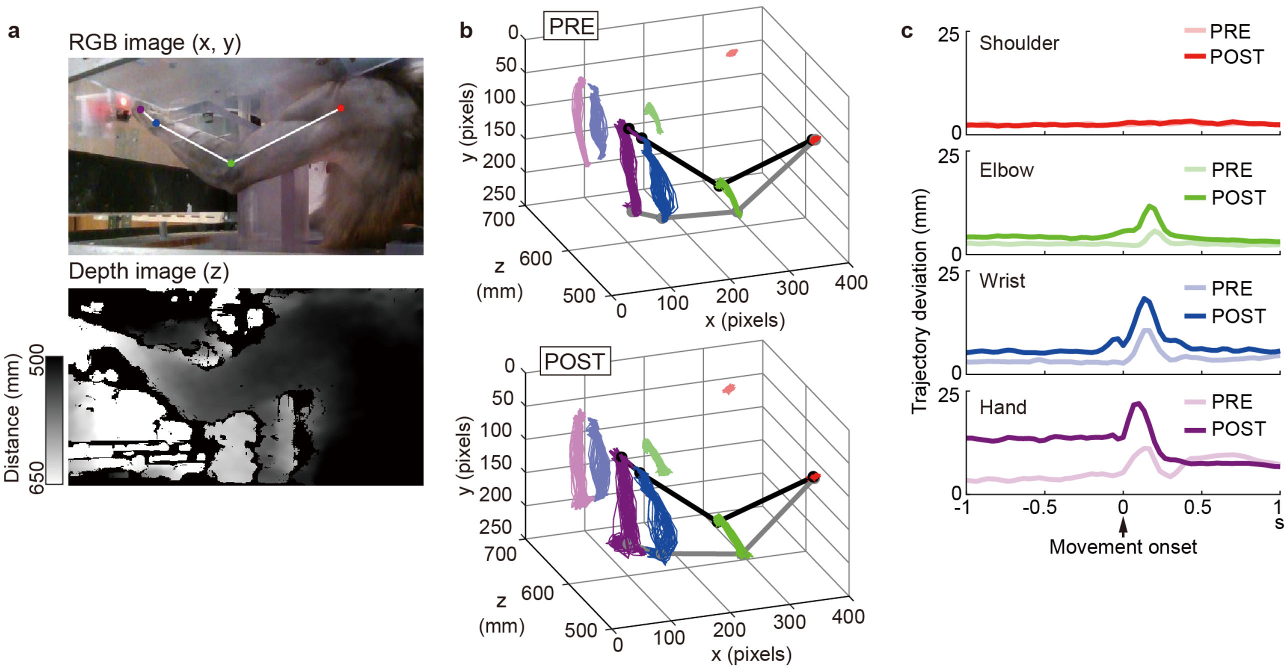

Supplementary Fig. 4 | Trajectory analysis of arm joints during the task. a, Example of RGB (x-y) and depth (z) images of monkey K captured using a depth camera. The RGB image was processed using DeepLabCut to detect the shoulder (red circle), elbow (green), wrist (blue), and hand (purple). **b**, Example of 3D trajectories of the shoulder (red), elbow (green), wrist (blue), and hand (purple) from −1.0 to +1.0 s relative to Movement onset and mean positions at −1.0 s (gray sticks) and +1.0 s (black sticks) in ET trials of monkey K in the PRE and POST periods. Trajectories were projected on the x-y plane using pale colors. **c**, Trajectory deviations, the differences from the mean trajectories, of each arm part during movements in the PRE and POST periods.

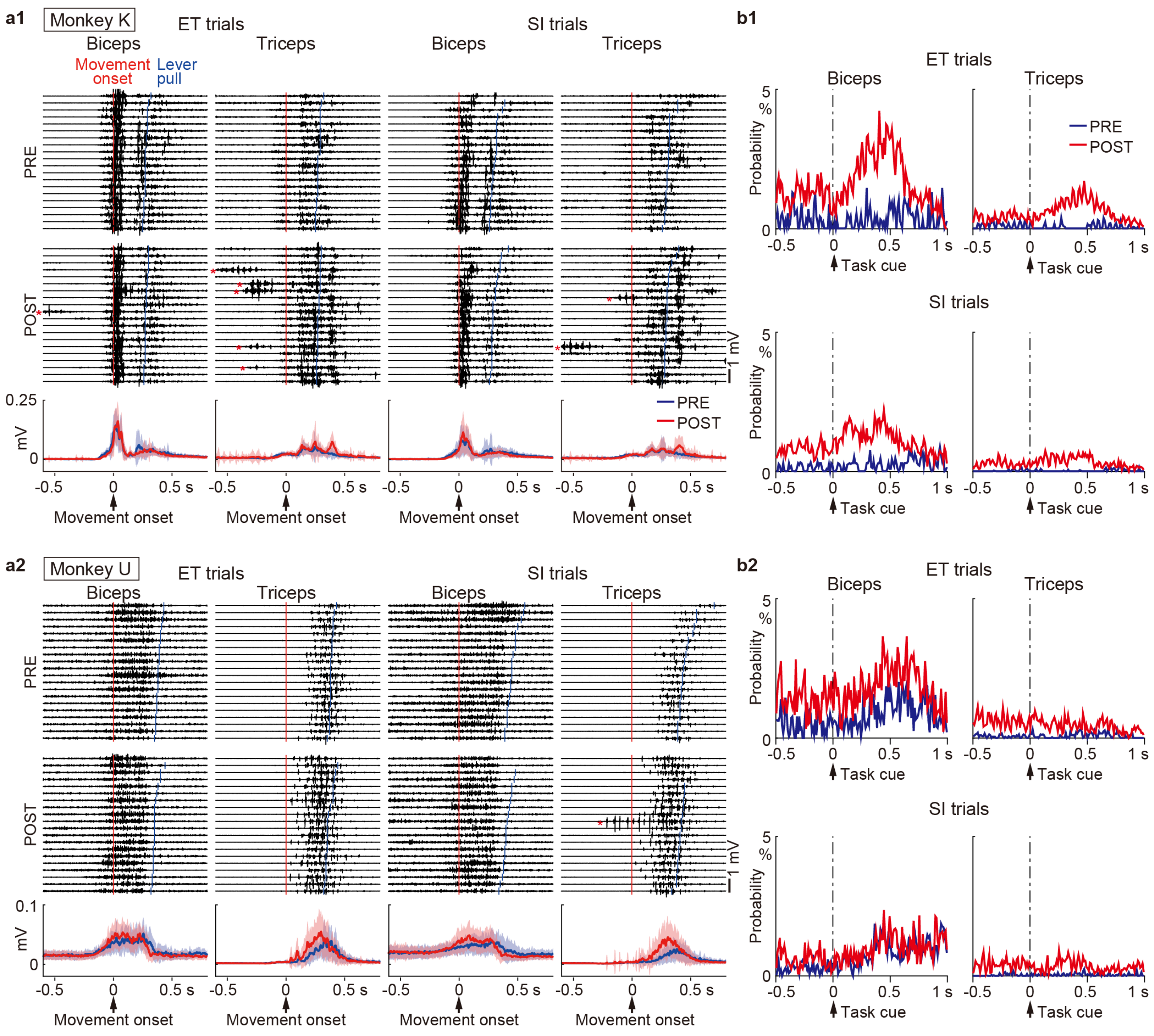

Supplementary Fig. 5 | EMGs of the contralateral forelimbs of monkeys K and U during the task. a, Raw EMGs and root mean square (RMS) of EMGs in a single session for monkey K (**a1**) and monkey U (**a2**), aligned with Movement onset in the PRE period (from −15 to 0 min) and POST period (from 10 to 45 min after DCZ administration). Examples of 20 EMG traces are sorted by MT (top two rows). Red and blue vertical lines indicate the timings of Movement onset and Lever pull, respectively. The RMS of EMGs was averaged in each period in 1-ms bins (mean ± SD; bottom). Red asterisks indicate task-irrelevant muscle activity, presumably corresponding to involuntary movements. Shading indicates SEM. **b**, Occurrence of abnormal EMG events before and after Task cue for monkey K (**b1**) and monkey U (**b2**). An abnormal EMG event was defined as an RMS increase above the mean + 3SD of the baseline activity (during the 500 ms before Task cue). Bin width, 10 ms. To calculate the occurrence probability, EMG recordings for 3 and 4 days were combined for monkeys K and U, respectively.

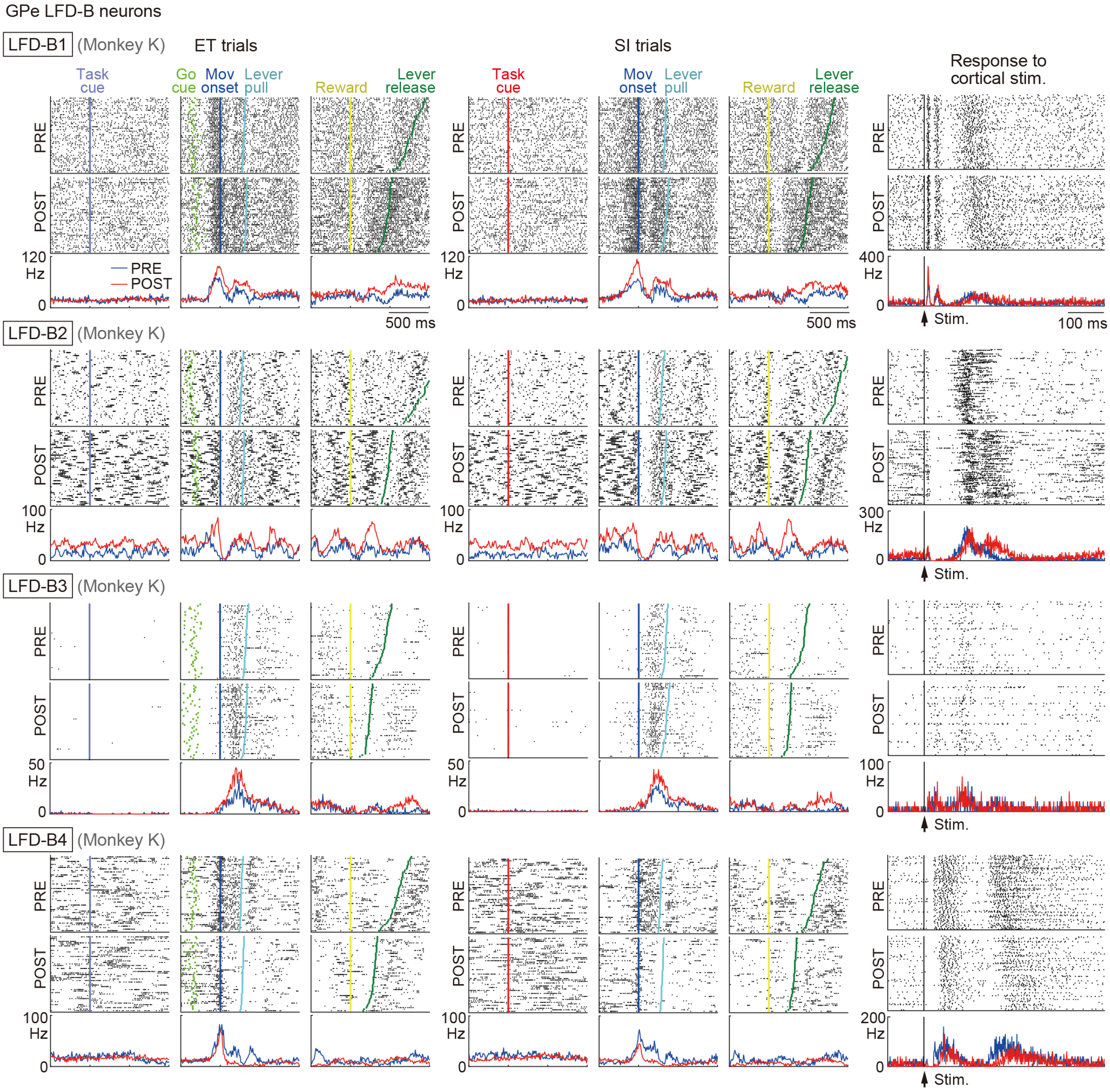

Supplementary Fig. 6 | Raster plots, PETHs, and PSTHs to cortical stimulation of LFD-B neurons in the GPe. Raster plots, PETHs, and PSTHs of all LFD-B neurons in the GPe of monkey K. Spikes are aligned with Task cue, Movement onset (Mov onset), and Reward delivery in the ET and SI trials. Raster plots are sorted by the time to the next task event. Bin width, 10 ms. Four LFD-B neurons (1-4) responded to cortical stimulation (triphasic, inhibition, or excitation) and exhibited task-related activity. After reduction of STN activity, task-related activity was generally enhanced (LFD-B1-3), and a burst firing pattern emerged. Further analysis of LFD-B neurons was not performed because of their small number. LFD-B and HFD-P neurons are considered to correspond to arkypallidal and prototypic neurons reported in the GPe of rodents[4].

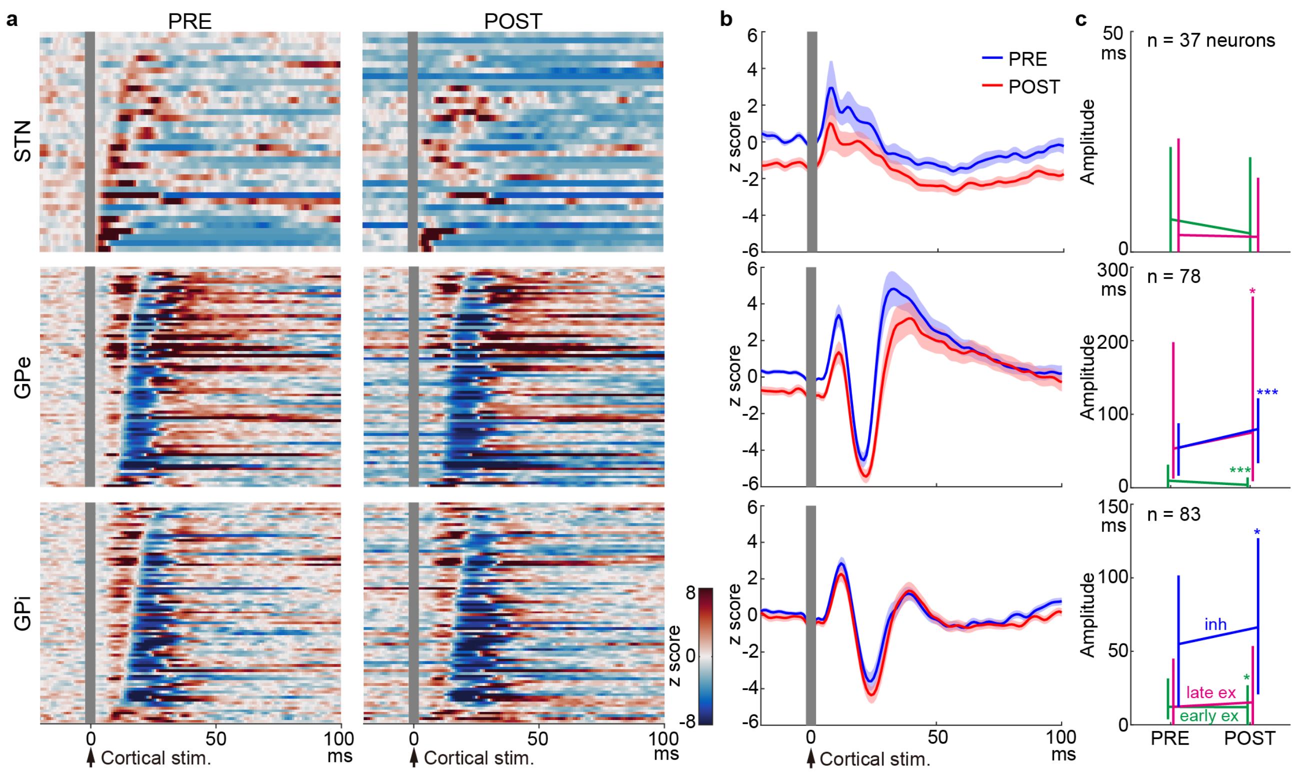

Supplementary Fig. 7 | Cortically evoked responses of STN/GPe/GPi neurons in the PRE and POST periods. a, Heatmaps of PSTHs of all 37 STN (from monkeys K and U), 78 GPe (from monkeys K and U), and 83 GPi (from monkeys E, K, and U) neurons in the PRE and POST periods. Each PSTH (bin width, 1 ms) was constructed from 100 repetitions of cortical stimulation, smoothed with a Gaussian filter (σ = 1.6 ms), and converted to z-scores using the baseline activity during the 100-ms period preceding stimulation in the PRE period. Neurons were sorted by the latency of earliest response in the PRE period for the STN and latency of inhibition for the GPe/GPi. Stimulation artefacts were covered by gray vertical bars at $|t| < 2$ ms. **b**, Population-averaged PSTHs of all STN/GPe/GPi neurons. Solid lines and shading indicate mean and SEM. **c**, Change in amplitude of early excitation (early ex) and late excitation (late ex) in STN neurons and early ex, inhibition (inh), and late ex in GPe/GPi neurons. Amplitude was defined as the area between the z-scored PSTH and z = 0 during each significant response. Data are presented as medians with the 25th and 75th percentiles. The baseline firing rate during the 100 ms preceding stimulation significantly decreased in GPe (from 76.5 ± 25.6 Hz to 65.6 ± 26.7 Hz, mean ± SD, $P < 10^{-6}$, two-tailed Wilcoxon signed rank test) but not GPi (from 85.0 ± 29.1 Hz to 82.0 ± 29.1 Hz, P = 0.1) neurons. In the GPe, early ex ($P = 3 \times 10^{-5}$) decreased, and inh ($P = 2 \times 10^{-8}$) and late ex (P = 0.03) increased; in the GPi, early ex (P = 0.04) decreased, and inh (P = 0.02) increased. * $P < 0.05$, ** $P < 0.01$, *** $P < 0.001$.

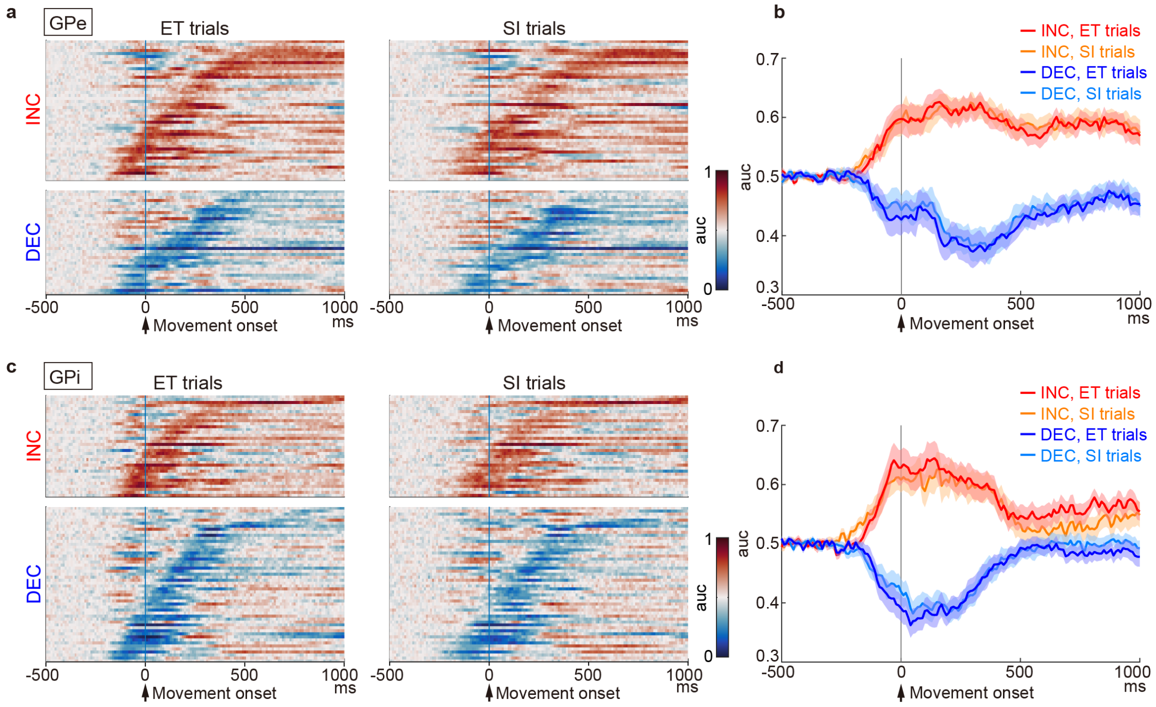

Supplementary Fig. 8 | Movement-related activity of GPe/GPi neurons in ET and SI trials in the PRE period. a, Heatmaps for 78 GPe neurons classified as 43 activity increase (INC) and 35 activity decrease (DEC) type neurons during movements. Firing rates were converted to area under curve (auc) of ROC analysis. Neurons are sorted by the onset of movement-related activity in ET trials (left). The activity of the same neuron in SI trials is shown on the same row (right). Bin width, 10 ms. **b,** Population-averaged PETHs of INC- and DEC-type GPe neurons in the ET and ST trials. Solid lines and shading indicate mean and SEM, respectively. **c, d**, Same as **(a, b)** but for 83 GPi neurons classified as 33 INC- and 50 DEC-type neurons. Solid lines and shading indicate mean and SEM, respectively (**b**, **d**).

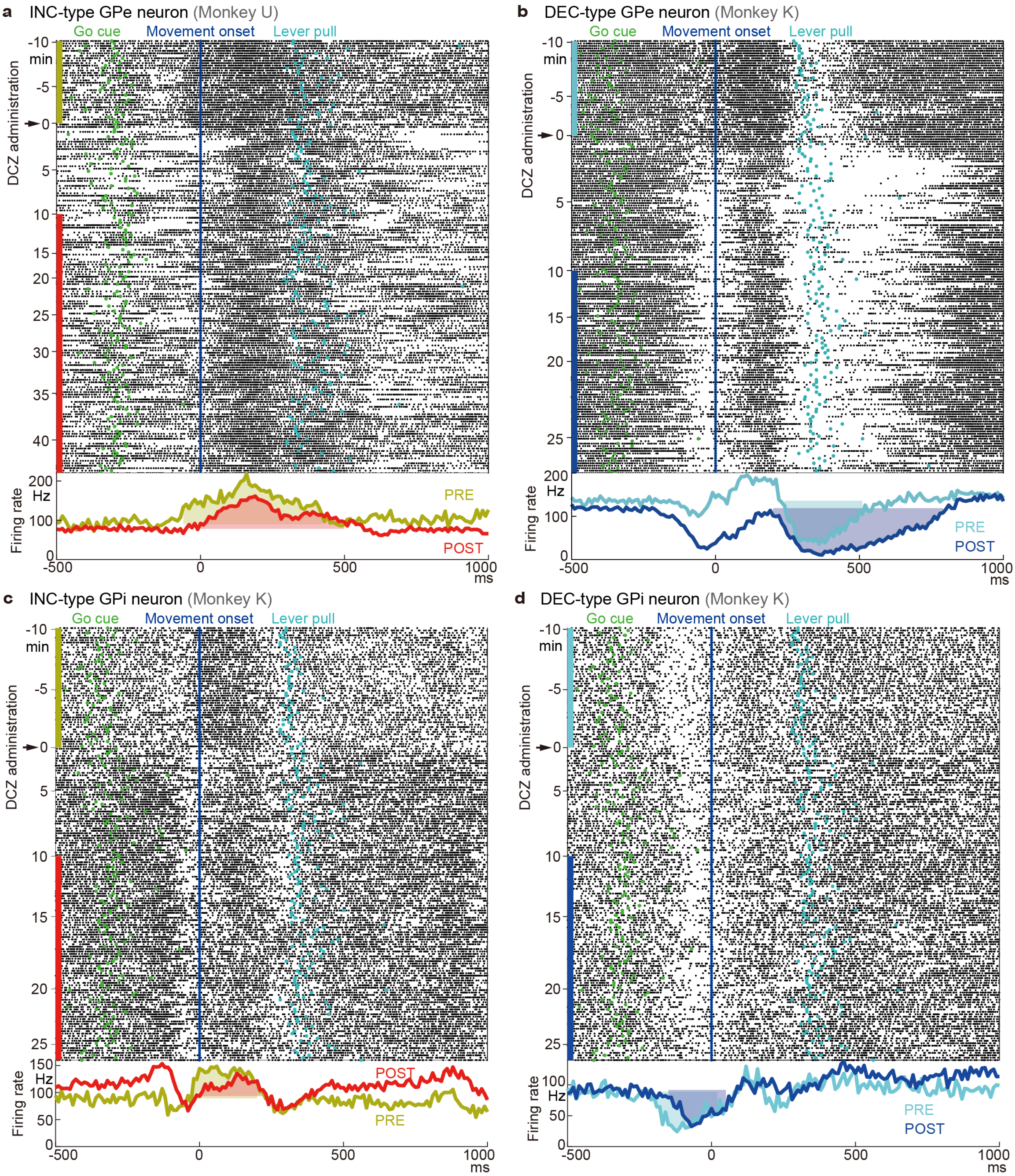

Supplementary Fig. 9 | Examples of movement-related activity of GPe/GPi neurons in ET trials. a, Example of a GPe neuron exhibiting an activity increase (INC) during movements. Raster plot aligned with Movement onset is displayed chronologically by elapsed time during the experiment (top); vertical colored bars on the left indicate the PRE and POST periods. The timings of Go cue and Lever pull are also indicated. PETHs in the PRE and POST periods are plotted with different colors (bottom; 10-ms bins), with shading indicating significant movement-related modulations. **b**, Another GPe neuron demonstrating an activity decrease (DEC). **c**, **d**, Same as (**a**, **b**) but for GPi neurons.

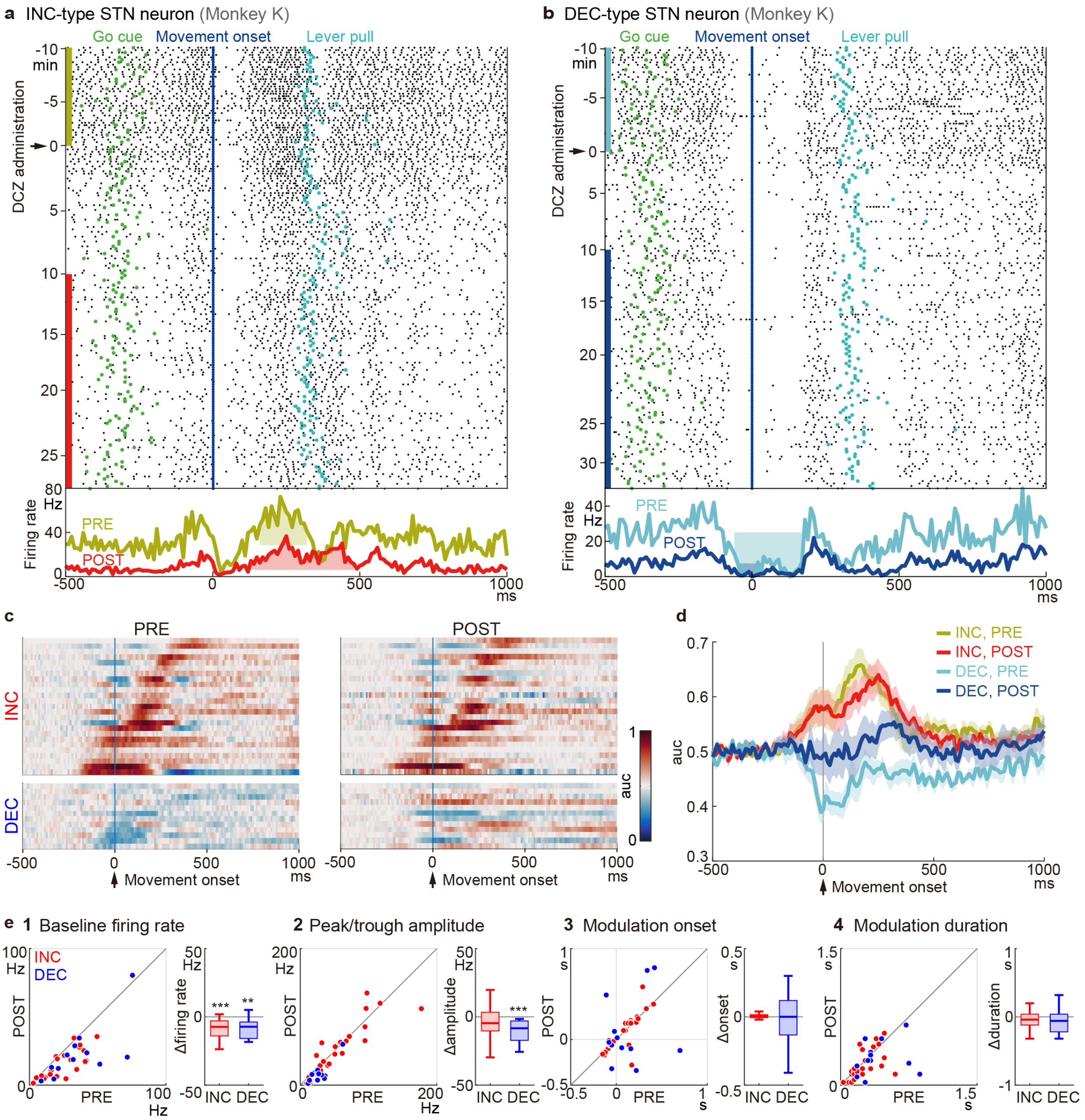

Supplementary Fig. 10 | Changes in movement-related activity of STN neurons after reduction of STN activity in ET trials. a, Example of a STN neuron exhibiting an activity increase (INC) during movements. **b**, Another STN neuron exhibiting an activity decrease (DEC). **c**, Heatmaps for 37 STN neurons classified as 25 INC- and 12 DEC-type neurons in the PRE and POST periods. **d**, Population-averaged PETHs. Solid lines and shading indicate mean and SEM, respectively. **e**, Scatter and box (an inner horizontal line, median; box, 25th and 75th percentiles; whiskers, maximum and minimum values within 1.5 times the interquartile range from the upper and lower quartiles) plots of change in PETHs between the PRE and POST periods of 25 INC- and 12 DEC-type neurons. Baseline firing rates of both INC- ($P = 4 \times 10^{-5}$, two-tailed Wilcoxon signed rank test) and DEC- ($P = 0.003$) type neurons decreased significantly. On the other hand, movement-related activity was affected only weakly: Trough amplitude in DEC-type neurons was decreased ($P = 0.0004$), while other parameters showed no significant changes. * $P < 0.05$, ** $P < 0.01$, *** $P < 0.001$.

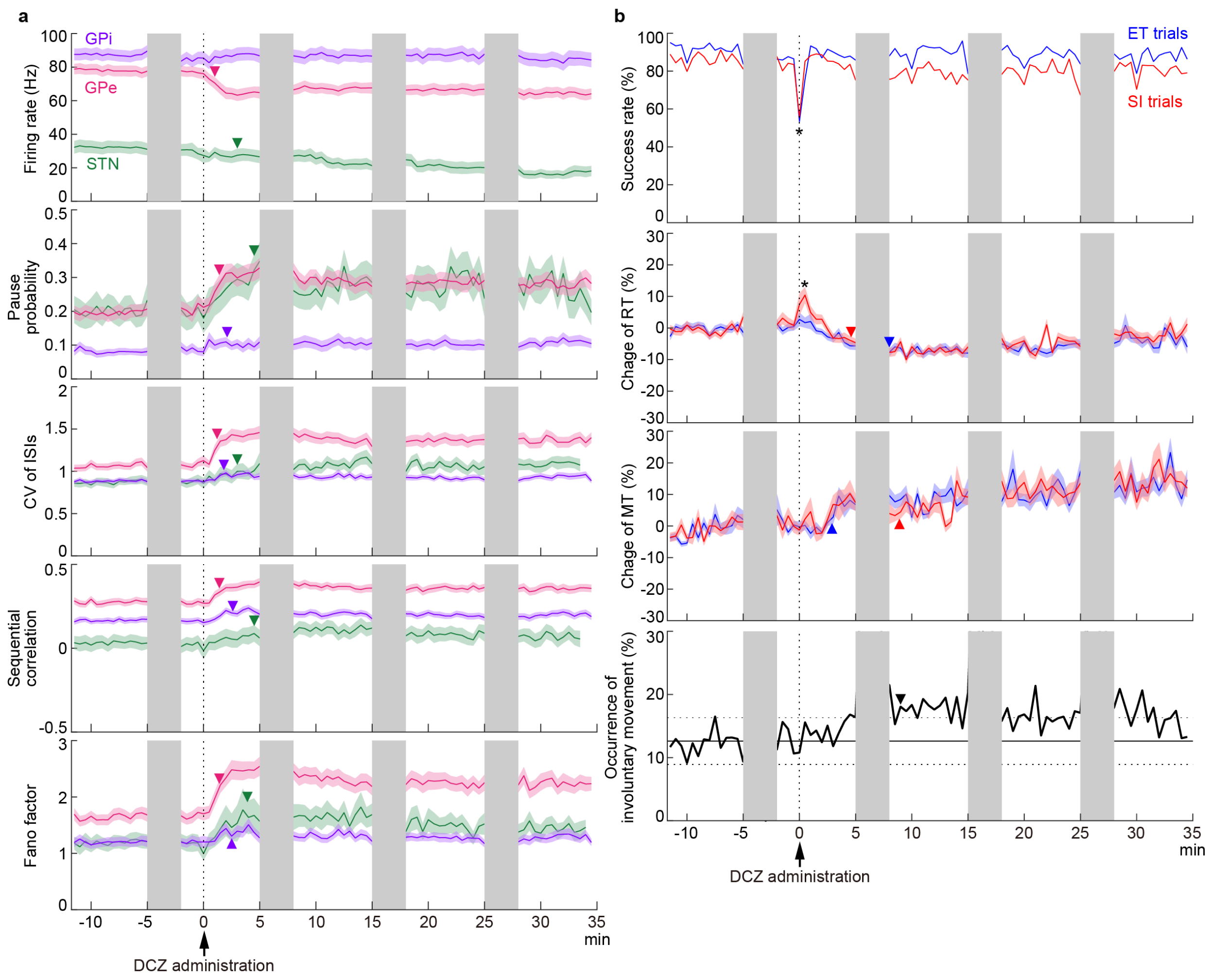

Supplementary Fig. 11 | Time course of changes in firing pattern of STN/GPe/GPi neurons and task performance with DCZ administration. a, Firing rate, Pause probability, CV of ISIs, Sequential correlation, and Fano factor in 37 STN, 78 GPe, and 63 GPi neurons of monkeys K and U were calculated during the period from −1.0 to 1.0 s relative to Movement onset in both the ET and SI trials. Solid lines and shading indicate population averages and SEM, respectively. Bin width, 30 s. In each neuron, firing patterns in every 10 trials were compared with those in the PRE period using the two-tailed Mann-Whitney U test. Onset of firing rate and pattern modulations in each neuron was defined as the timing of the first trial in two consecutive trial sets (i.e., 20 trials) with $P < 0.05$; the timings at which half of modulated neurons showed significant changes are indicated by arrowheads. Firing rate modulation in the GPi was not significant (Fig. 4b). The periods of cortical stimulation are indicated by gray shading and excluded from the analysis. **b,** Success rate, Changes of RT (percentage change from mean RT in the PRE period), and Changes of MT (percentage change from mean MT in the PRE period) of ET and SI trials, and Occurrence of involuntary movement (before Task cue, Fig. 2e) were calculated from 84 sessions of monkeys K and U. Bin width, 30 s. Onsets of RT and MT changes were defined in the same method used above. Onset of occurrence of involuntary movement was defined as the first of two consecutive bins outside the baseline probability (mean ± 1.96SD in the PRE period; horizontal solid and dotted lines). *, Transient changes of Success rate and RT are presumably due to the distraction by the intramuscular injection of DCZ.

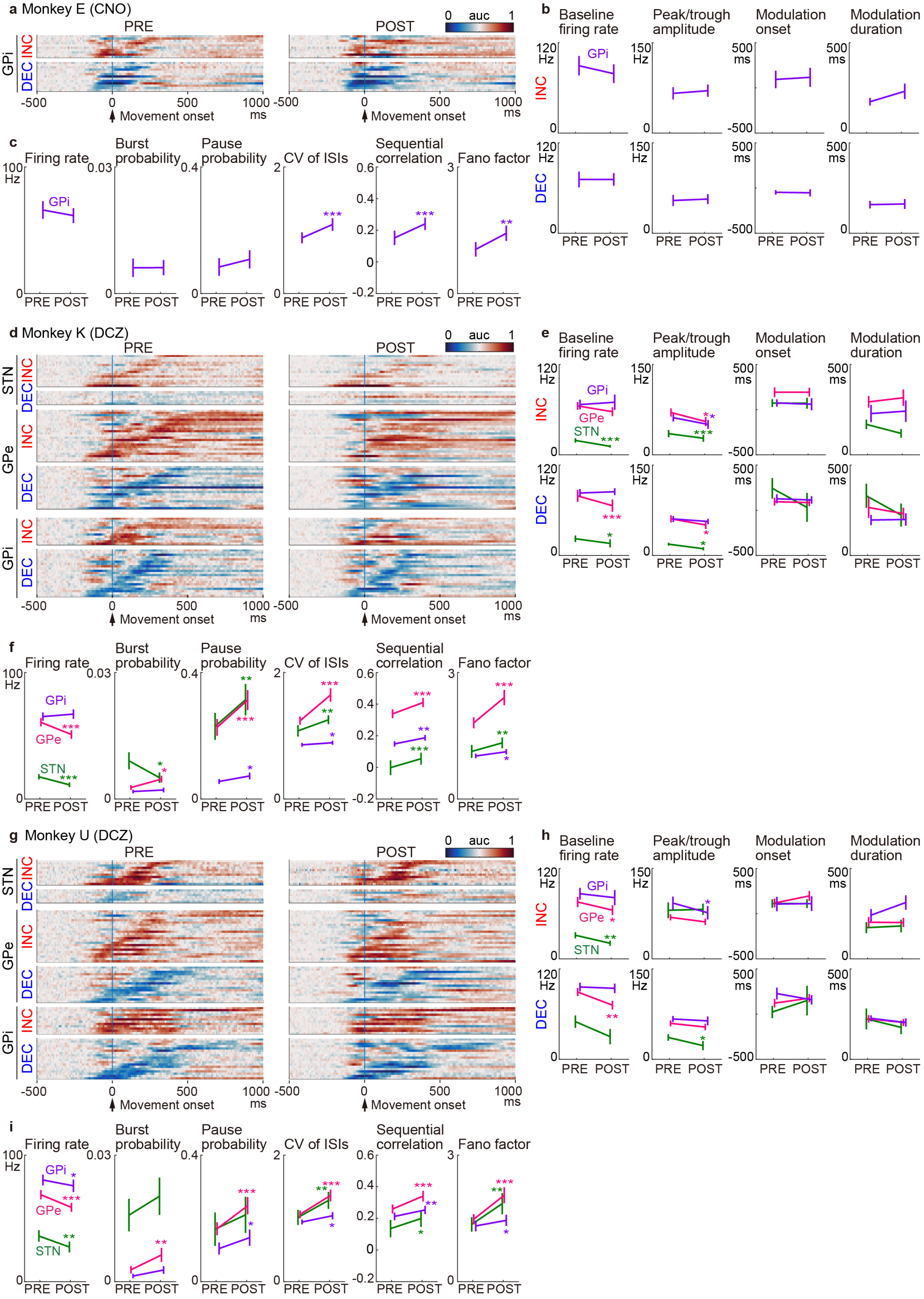
a Monkey E (CNO)
PRE
POST
0 auc 1
GPi
INC
DEC
-500
0
500
1000
ms
Movement onset
b
Baseline firing rate
Peak/trough amplitude
Modulation onset
Modulation duration
120 Hz
150 Hz
500 ms
-500
GPi
PRE POST
c
Firing rate
Burst probability
Pause probability
CV of ISIs
Sequential correlation
Fano factor
100 Hz
0.03
0.4
2
0.6
0.2
-0.2
3
***
**
d Monkey K (DCZ)
STN
GPe
e
GPe
STN
f
g Monkey U (DCZ)
h
i

Supplementary Fig. 12 | Neuronal activity changes of STN/GPe/GPi neurons after reduction of STN activity in each animal. a, Heatmaps for 20 GPi neurons classified as 9 INC- (45%) and 11 DEC- (55%) type neurons of monkey E in the PRE and POST periods with CNO administration. **b,** Changes in PETHs between the PRE and POST periods of 9 INC and 11 DEC types of GPi neurons. Details follow those of Figure 3c. **c,** Statistical analyses of spike trains of 20 GPi neurons. CV of ISIs, Sequential correlation, and Fano factor (P = 0.0003, 0.0006, and 0.003, respectively; two-tailed Wilcoxon signed rank test) increased. Details follow those of Figure 4b. **d-f**, Same as (**a-c)** but for 20 STN neurons classified as14 INC- (70%) and 6 DEC- (30%) type neurons, 39 GPe neurons classified as 20 INC- (51%) and 19 DEC- (49%) type neurons, and 33 GPi neurons classified as 12 INC- (36%) and 21 DEC- (64%) type neurons of monkey K with DCZ administration. Baseline firing rate of INC-type STN, DEC-type STN, and DEC-type GPe neurons (P = 0.0009, 0.03, and 0.0005), and Peak/trough amplitude of INC-type STN, INC-type GPe, INC-type GPi, DEC-type STN, and DEC-type GPe (0.0004, 0.01, 0.03, 0.03, and 0.02) decreased. Firing rate of STN and GPe neurons (9×10^{-5} and 3×10^{-4}) and Burst probability of STN (0.04) decreased. Burst probability of GPe neurons (0.03), Pause probability of STN, GPe, and GPi neurons (0.002, 7×10^{-5}, and 0.02), CV of ISIs of STN, GPe, and GPi neurons (0.004, 5×10^{-7}, and 0.04), Sequential correlation of STN, GPe, and GPi neurons (0.0008, 3×10^{-5}, and 0.003), and Fano factor of of STN, GPe, and GPi neurons (0.001, 4×10^{-6}, and 0.03) increased. **g-i**, Same as (**a-c)** but for 17 STN neurons classified as 11 INC- (65%) and 6 DEC- (35%) type neurons, 39 GPe neurons classified as 23 INC- (59%) and 16 DEC- (41%) type neurons, and 30 GPi neurons classified as 12 INC- (40%) and 18 DEC- (60%) type neurons of monkey U with DCZ administration. Baseline firing rates of INC-type STN, INC-type GPe, and DEC-type GPe neurons (0.003, 0.02, and 0.006) and Peak/trough amplitude INC-type GPi and DEC-type STN neurons (0.02 and 0.03) decreased. Firing rate of STN, GPe, and GPi neurons (0.002, 0.0003, and 0.03) decreased. Burst probability of GPe neurons (0.005), Pause probability of GPe and GPi neurons (4×10^{-5} and 0.03), CV of ISIs of STN, GPe, and GPi neurons (0.005, 7×10^{-6}, and 0.01), Sequential correlation of STN, GPe, and GPi neurons (0.02, 1×10^{-6}, and 0.009), and Fano factor of STN, GPe, and GPi neurons (0.006, 3×10^{-6}, and 0.05) increased. Error bars indicate SEM (**b**, **c**, **e**, **f**, **h**, **i**). * $P < 0.05$, ** $P < 0.01$, *** $P < 0.001$.

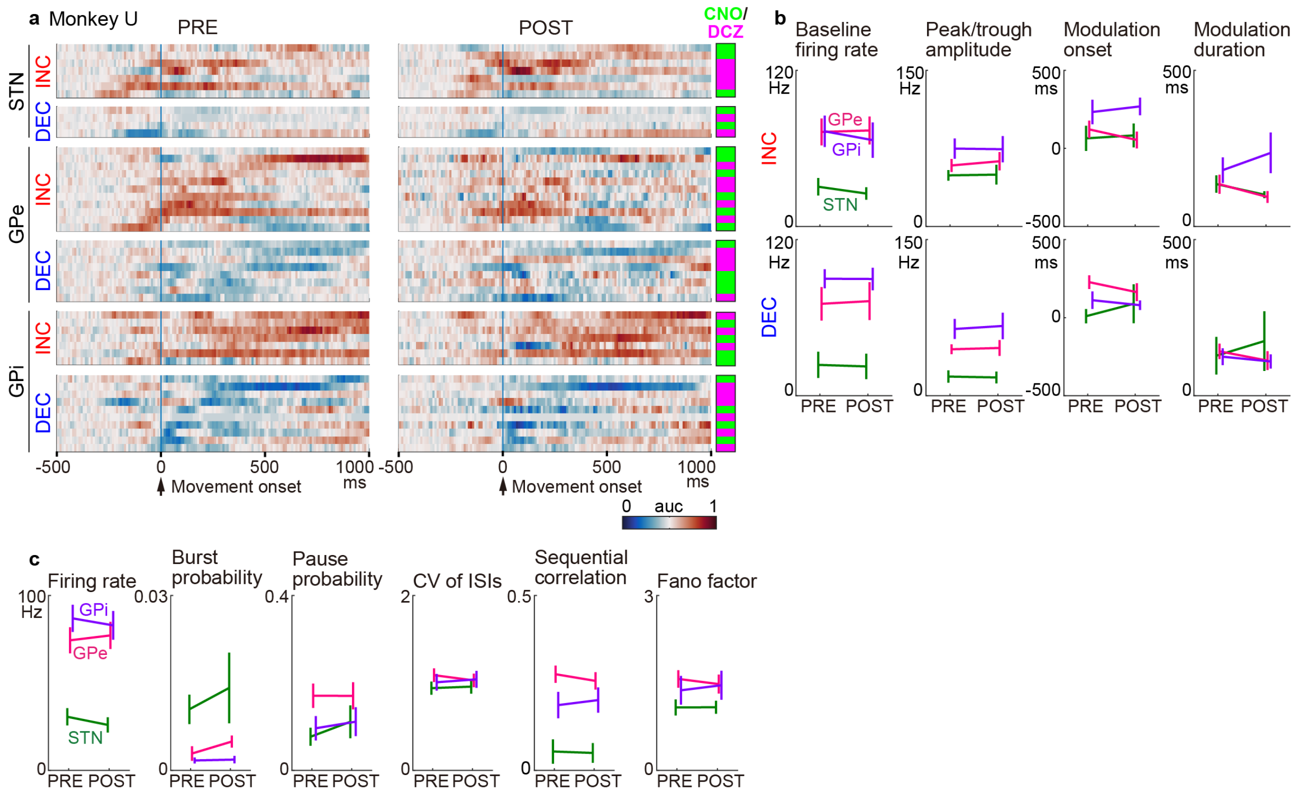

Supplementary Fig. 13 | Movement-related activity and firing rate/pattern of STN/GPe/GPi neurons in the AAV non-injection side. Single unit activity was recorded from the STN/GPe/GPi in the AAV non-injection side, while a monkey performed the same reaching task using the hand contralateral to the recording side. **a,** Heatmaps for 11 STN (7 INC and 4 DEC types), 19 GPe (11 INC and 8 DEC types), and 16 GPi (7 INC and 9 DEC types) neurons of monkey U in the PRE and POST periods with CNO (1.0 mg/kg, i.v.) or DCZ (0.1 mg/kg, i.m.) administration (indicated by color barcodes in the right). **b,** Changes in PETHs of 11 STN, 19 GPe, and 16 GPi neurons between the PRE and POST periods. Error bars indicate SEM. Details follow those of Figure 3c. **c,** Changes in spike trains of 11 STN, 19 GPe, and 16 GPi neurons. Error bars indicate SEM. Details follow those of Figure 4b. No significant changes in the two-tailed Wilcoxon signed rank test.

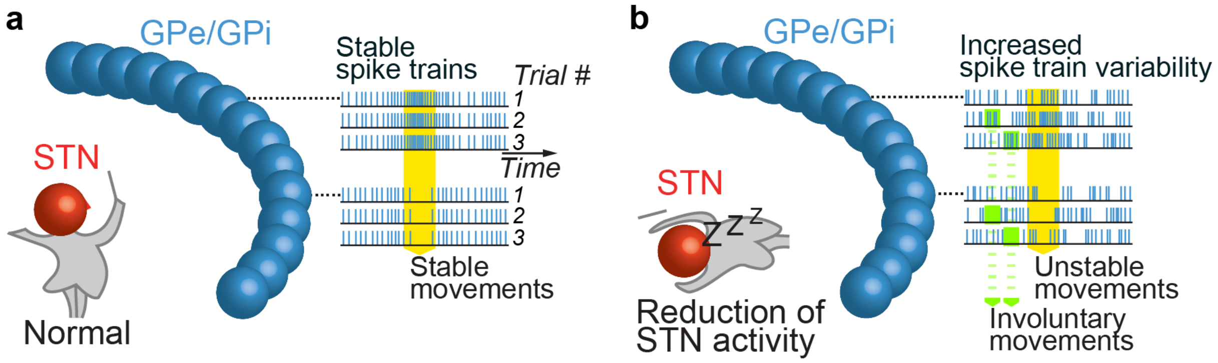

Supplementary Fig. 14 | Hypothetical role of the STN on motor control. a, Neural activity of the BG in the normal state. The STN interacts with the GPe/GPi to generate stable activity in the resting state and coordinated firing rate changes during movements (yellow), contributing to suppression of involuntary movements and stable movements, respectively. **b**, Neural activity during the reduction of STN activity. Spike trains of GPe/GPi neurons become variable. Increased spike train variability during movements (yellow) leads to unstable movements. In the resting state, increased spike train variability may increase the chance of coincident activity changes (green) similar to those during voluntary movements, resulting in involuntary movements.

Supplementary Table 1 | Summary of manipulation of the STN in normal sub-human primates

References	Method	Target	% of STN affected	Motor signs	Neural activity change
Whittier and Mettler 1949[5]	Electrolytic lesion	STN	20-60% (of volume)	Hyperkinesia in upper and lower limbs	N/A
Carpenter et al. 1950[6]	Electrolytic lesion	STN	20-70% (of volume)	Hyperkinesia in upper and lower limbs	N/A
Crossman et al. 1984[7] Mitchell et al. 1985[8]	Injection of $GABA_A$ receptor antagonist	STN	N/A	Dyskinesia in upper and lower limbs	Decrease of 2-deoxyglucose uptake in GPe/GPi.
Hamada and Delong 1992[9,10]	Excitotoxic lesion	STN	30-50% (of volume)	Dyskinesia in upper and lower limbs	Decreased firing rates in GPe/GPi.
Beurrier et al. 1997[11]	High frequency electric stimulation	STN	N/A	Dyskinesia in upper and lower limbs	N/A
Nambu et al. 2000[12]	Injection of $GABA_A$ receptor agonist	STN (sensorimotor region)	N/A	Hemiballism in upper and lower limbs	Decreased firing rates in GPe/GPi. Reduced cortically induced excitation and enhanced cortically induced inhibition in GPe/GPi
Karachi et al. 2009[13]	Injection of $GABA_A$ receptor agonist/antagonist	STN (sensorimotor and associative/limbic regions)	N/A	Hemiballism in upper and lower limbs, head deviation (sensorimotor region) Stereotypic behaviors and violent hyperactivity (associative/limbic region)	N/A
Present study	Chemogenetic inhibition	STN (sensorimotor region)	20-30% (of neurons)	Hyperkinesia in upper limb	Decreased firing rates in GPe. Increased spike train variability in GPe/GPi.

Supplementary References

1. Vitek, J. L., Ashe, J., DeLong, M. R. & Alexander, G. E. Physiologic properties and somatotopic organization of the primate motor thalamus. *J. Neurophysiol.* **71**, 1498–1513 (1994).
2. Vitek, J. L., Ashe, J., DeLong, M. R. & Kaneoke, Y. Microstimulation of primate motor thalamus: somatotopic organization and differential distribution of evoked motor responses among subnuclei. *J. Neurophysiol.* **75**, 2486–2495 (1996).
3. Nambu, A. Somatotopic organization of the primate Basal Ganglia. *Front. Neuroanat.* **5**, 26 (2011).
4. Mallet, N. *et al.* Dichotomous organization of the external globus pallidus. *Neuron* **74**, 1075–1086 (2012).
5. Whittier, J. R. & Mettler, F. A. Studies on the subthalamus of the rhesus monkey; hyperkinesia and other physiologic effects of subthalamic lesions; with special reference to the subthalamic nucleus of Luys. *J. Comp. Neurol.* **90**, 319–372 (1949).
6. Carpenter, M. B., Whittier, J. R. & Mettler, F. A. Analysis of choreoid hyperkinesia in the Rhesus monkey; surgical and pharmacological analysis of hyperkinesia resulting from lesions in the subthalamic nucleus of Luys. *J. Comp. Neurol.* **92**, 293–331 (1950).
7. Crossman, A. R., Sambrook, M. A. & Jackson, A. Experimental hemichorea/hemiballismus in the monkey. Studies on the intracerebral site of action in a drug-induced dyskinesia. *Brain* **107**, 579–596 (1984).
8. Mitchell, I. J., Sambrook, M. A. & Crossman, A. R. Subcortical changes in the regional uptake of [3H]-2-deoxyglucose in the brain of the monkey during experimental choreiform dyskinesia elicited by injection of a gamma-aminobutyric acid antagonist into the subthalamic nucleus. *Brain* **108**, 405–422 (1985).
9. Hamada, I. & DeLong, M. R. Excitotoxic acid lesions of the primate subthalamic nucleus result in transient

dyskinesias of the contralateral limbs. *J. Neurophysiol.* **68**, 1850–1858 (1992).

10. Hamada, I. & DeLong, M. R. Excitotoxic acid lesions of the primate subthalamic nucleus result in reduced pallidal neuronal activity during active holding. *J. Neurophysiol.* **68**, 1859–1866 (1992).

11. Beurrier, C., Bezard, E., Bioulac, B. & Gross, C. Subthalamic stimulation elicits hemiballismus in normal monkey. *Neuroreport* **8**, 1625–1629 (1997).

12. Nambu, A. *et al.* Excitatory cortical inputs to pallidal neurons via the subthalamic nucleus in the monkey. *J. Neurophysiol.* **84**, 289–300 (2000).

13. Karachi, C. *et al.* Dysfunction of the subthalamic nucleus induces behavioral and movement disorders in monkeys. *Mov. Disord.* **24**, 1183–1192 (2009).
