# Supplementary material for: Subthalamic nucleus stabilizes movements by reducing neural spike variability in monkey basal ganglia
Source: Nat Commun. 2022 Apr 25;13:2233. doi: 10.1038/s41467-022-29750-2 (PMC9038919; doi:10.1038/s41467-022-29750-2)
Supplement: Supplementary file 4 — Reporting Summary [file 41467_2022_29750_MOESM4_ESM.pdf]

nature portfolio

Corresponding author(s): Atsushi Nambu

Last updated by author(s): Mar 10, 2022

Reporting Summary

Nature Portfolio wishes to improve the reproducibility of the work that we publish. This form provides structure for consistency and transparency in reporting. For further information on Nature Portfolio policies, see our Editorial Policies and the Editorial Policy Checklist.

Statistics

For all statistical analyses, confirm that the following items are present in the figure legend, table legend, main text, or Methods section.

n/a	Confirmed	
☐	☒	The exact sample size (n) for each experimental group/condition, given as a discrete number and unit of measurement
☐	☒	A statement on whether measurements were taken from distinct samples or whether the same sample was measured repeatedly
☐	☒	The statistical test(s) used AND whether they are one- or two-sided<br>*Only common tests should be described solely by name; describe more complex techniques in the Methods section.*
☐	☒	A description of all covariates tested
☐	☒	A description of any assumptions or corrections, such as tests of normality and adjustment for multiple comparisons
☐	☒	A full description of the statistical parameters including central tendency (e.g. means) or other basic estimates (e.g. regression coefficient) AND variation (e.g. standard deviation) or associated estimates of uncertainty (e.g. confidence intervals)
☐	☒	For null hypothesis testing, the test statistic (e.g. F, t, r) with confidence intervals, effect sizes, degrees of freedom and P value noted<br>*Give P values as exact values whenever suitable.*
☒	☐	For Bayesian analysis, information on the choice of priors and Markov chain Monte Carlo settings
☒	☐	For hierarchical and complex designs, identification of the appropriate level for tests and full reporting of outcomes
☐	☒	Estimates of effect sizes (e.g. Cohen's d, Pearson's r), indicating how they were calculated

Our web collection on statistics for biologists contains articles on many of the points above.

Software and code

Policy information about availability of computer code

Data collection	The behavioral task was controlled and logged with a custom script written in LabVIEW 2013 (National Instruments). In the 3D trajectory analyses, a depth camera was controlled with a custom script written in MATLAB R2019b (MathWorks). Electrophysiologic signals were recorded with AlphaLab SnR v2.0.0 (Alpha Omega). Immunohistologic images were taken with BZ-X Viewer v1.3.2 (Keyence).
Data analysis	DeepLabCut v2.0.7 (https://github.com/DeepLabCut/DeepLabCut) was used to reconstruct the trajectories for arm joints. Custom scripts written in MATLAB R2019b (MathWorks) were used for all other analyses. Custom MATLAB scripts used to analyze the electrophysiological signals and the 3D trajectory for arm joints are available at Zenodo: https://doi.org/10.5281/zenodo.6346369.

For manuscripts utilizing custom algorithms or software that are central to the research but not yet described in published literature, software must be made available to editors and reviewers. We strongly encourage code deposition in a community repository (e.g. GitHub). See the Nature Portfolio guidelines for submitting code & software for further information.

Data

Policy information about availability of data

All manuscripts must include a data availability statement. This statement should provide the following information, where applicable:

- Accession codes, unique identifiers, or web links for publicly available datasets
- A description of any restrictions on data availability
- For clinical datasets or third party data, please ensure that the statement adheres to our policy

Data files for neural spike trains that support the findings of this study are available at Zenodo: https://doi.org/10.5281/zenodo.6340403. Data files for behavioral tests and raw electrophysiological signals are too large for an online repository, and they are available from the corresponding author upon reasonable request.

Field-specific reporting

Please select the one below that is the best fit for your research. If you are not sure, read the appropriate sections before making your selection.

☒ Life sciences ☐ Behavioural & social sciences ☐ Ecological, evolutionary & environmental sciences

For a reference copy of the document with all sections, see nature.com/documents/nr-reporting-summary-flat.pdf

Life sciences study design

All studies must disclose on these points even when the disclosure is negative.

Sample size	Three macaque monkeys were included in this study. All three monkeys underwent the following experimental procedures: AAV injection, behavioral tests, and electrophysiologic recordings. Behavioral effects of DREADD ligands were analyzed within each individual for all three monkeys. Histological assay was performed in two monkeys. The sample size of three monkeys was selected to balance between the repeatability and cost of experiments and commonly used in electrophysiologic experiments with non-human primates. In the analyses of neural activity, 37 STN neurons from two monkeys, 78 GPe neurons from two monkeys, and 83 GPi neurons from three monkeys were used. No statistical methods were used to pre-determine the number of neurons to analyze, but the significance of our major findings was confirmed by 1) analyzing neural units in each monkey (Supplementary Fig. 12) and 2) recording neural signals in the AAV non-injected side from one monkey (Supplementary Fig. 13).
Data exclusions	Only neurons with isolation score ≥0.6 during the whole recording period were analyzed as described in Methods. In addition, neurons that do not respond to electrical stimulation on the motor cortices were excluded, since only the motor loop in the basal ganglia was focused in this study.
Replication	In each monkey, behavioral and/or electrophysiologic experiments was limited to once every other day. A DREADD ligand or vehicle was administered in a random manner. Similar abnormal behaviors were observed in all three monkeys in response to the administration of a DREADD ligand. Neural spike analyses performed on each monkey produced qualitatively similar results (Supplementary Fig. 12).
Randomization	While analyzing behavioral and EMG data, all comparisons were performed within each subject before and after the administration of a DREADD ligand or vechile. For electrophysiologic data, all comparisons were performed within each neuron (i.e., before and after the administration [Fig. 3, 4], between trials with short and long movement times [Fig. 5], or between trials with or without involuntary movements [Fig. 6]). Since no comparisons and statistical test were performed across subjects, randomization of subjects to treatment groups is not relevant. Treatments (i.e. administration of vechile or DREADD ligand) were randomized on a day-by-day basis during data collection.
Blinding	Blinding was not relevant to the present study, since no experimental groups were assigned.

Reporting for specific materials, systems and methods

We require information from authors about some types of materials, experimental systems and methods used in many studies. Here, indicate whether each material, system or method listed is relevant to your study. If you are not sure if a list item applies to your research, read the appropriate section before selecting a response.

Materials & experimental systems

n/a	Involved in the study
☐	☒ Antibodies
☐	☒ Eukaryotic cell lines
☒	☐ Palaeontology and archaeology
☐	☒ Animals and other organisms
☒	☐ Human research participants
☒	☐ Clinical data
☒	☐ Dual use research of concern

Methods

n/a	Involved in the study
☒	☐ ChIP-seq
☒	☐ Flow cytometry
☒	☐ MRI-based neuroimaging

Antibodies

Antibodies used	Rabbit anti-GFP antibody (1:1000, Invitrogen, A11122, polyclonal, Lot# 1925070)<br>Mouse anti-NeuN antibody (1:1000, Millipore, MAB377, monoclonal, clone A60, Lot# LV1634819)<br>Mouse anti-GFAP antibody (1:400, Sigma-Aldrich, G3893, Lot# 031M4836)<br>Goat anti-rabbit IgG conjugated with Alexa Fluor 488 (1:500, Invitrogen, A11043, polyclonal, Lot# 1735088)<br>Goat anti-mouse IgG conjugated with Alexa Fluor 594 (1:500, Invitrogen, A11032, polyclonal, Lot# 1922849)<br>Donkey anti-rabbit IgG, biotinylated (1:1000, Jackson, 711-065-152, Lot# 98352)
Validation	All commercial antibodies were validated by manufacturers, as follows:<br>Rabbit anti-GFP antibody: https://www.thermofisher.com/antibody/product/GFP-Antibody-Polyclonal/A-11122<br>Mouse anti-NeuN antibody: https://www.merckmillipore.com/INTL/en/product/Anti-NeuN-Antibody-clone-A60,MM_NF-MAB377

Mouse anti-GFAP antibody: https://www.sigmaaldrich.com/product/sigma/g3893
Goat anti-rabbit IgG antibody: https://www.thermofisher.com/antibody/product/Goat-anti-Rabbit-IgG-H-L-Cross-Adsorbed-Secondary-Antibody-Polyclonal/A-11008
Goat anti-mouse IgG antibody: https://www.thermofisher.com/antibody/product/Goat-anti-Mouse-IgG-H-L-Highly-Cross-Adsorbed-Secondary-Antibody-Polyclonal/A-11032
Donky anti-rabbit IgG antibody, biotinylated: https://www.jacksonimmuno.com/catalog/products/711-065-152

Eukaryotic cell lines

Policy information about cell lines

Cell line source(s)	Human Embryonic Kidney 293, cell line internally maintained, original source is American Type Culture Collection.
Authentication	Cells were authenticated by the vendor.
Mycoplasma contamination	Not tested.
Commonly misidentified lines (See ICLAC register)	None

Animals and other organisms

Policy information about studies involving animals; ARRIVE guidelines recommended for reporting animal research

Laboratory animals	One male and two female Japanese monkeys (Macaca fuscata; 5-8 kg, 4-7 years old)
Wild animals	No wild animals were used.
Field-collected samples	No field-collected samples were used.
Ethics oversight	The experimental protocols were approved by the Institutional Animal Care and Use Committee of the National Institutes of Natural Sciences.

Note that full information on the approval of the study protocol must also be provided in the manuscript.
